# Using Natural Language Processing to Examine State Child Maltreatment Policies and Associations With Outcomes: Multistate Cross-Sectional Study

**DOI:** 10.2196/99643

**Published:** 2026-09-16

**Authors:** Zhidi Luo, Richard A Epstein, Nethra Sambamoorthi, Lutfiyya N Muhammad, Neil Jordan

**Affiliations:** 1 Department of Psychiatry and Behavioral Sciences Northwestern University Feinberg School of Medicine Chicago, IL United States; 2 Health Sciences Integrated Program Northwestern University Feinberg School of Medicine Chicago, IL United States; 3 School of Professional Studies Northwestern University Chicago, IL United States; 4 Department of Preventive Medicine Northwestern University Feinberg School of Medicine Chicago, IL United States; 5 Department of Medical Social Sciences Northwestern University Feinberg School of Medicine Chicago, IL United States

**Keywords:** child abuse, child neglect, cluster analysis, clustering analysis, data mining, factor analysis, health policy, natural language processing, public policy

## Abstract

**Background:**

Child maltreatment is a major public health issue in the United States, with substantial variation in how states define, report, and respond to abuse and neglect. While prior research has examined individual policy components, less is known about how multiple policies jointly shape broader policy environments and relate to maltreatment outcomes.

**Objective:**

This study used natural language processing (NLP) to characterize state child maltreatment policy environments and examine their associations with maltreatment incidence, recurrence, and fatalities across US jurisdictions.

**Methods:**

A cross-sectional study was conducted using 2021 data from 50 US states, the District of Columbia, and Puerto Rico (N=52). A total of 411 state maltreatment policy items were derived from the State Child Abuse & Neglect Policies Database. A total of 6 NLP models (bidirectional and auto-regressive transformer [BART], bidirectional encoder representations from transformers [BERT], robustly optimized BERT approach [RoBERTa], decoding-enhanced BERT with disentangled attention [DeBERTa], Copilot, and LLaMA 3.1) were applied using a zero-shot classification framework to quantify policy characteristics. Models were evaluated using intrinsic (category consistency and semantic alignment) and extrinsic (factor analysis and clustering performance) metrics. Exploratory factor analysis was used to identify latent policy domains, and k-means clustering was applied to group jurisdictions with similar policy profiles. Maltreatment outcomes, including incidence, recurrence, and fatalities, were obtained from the National Child Abuse and Neglect Data System. Outcome differences across clusters were assessed using ANOVA with post hoc pairwise comparisons.

**Results:**

The study population included 72,838,819 children and 3,774,528 maltreatment reports, of which 751,283 were substantiated or indicated cases. Across 6 NLP models, DeBERTa demonstrated the best overall performance. Exploratory factor analysis identified 3 primary policy domains: maltreatment definition, mandated reporting, and alternative response. A total of 4 policy clusters were identified. Jurisdictions with weaker reporting requirements and fewer penalties (cluster 2) had the highest incidence of maltreatment (mean 17.51, SD 6.13 vs mean 9.65, SD 4.10; mean 10.28, SD 6.47; and mean 11.74, SD 6.47 per 1000 children; *P*=.07) and recurrence (mean 1.74, SD 0.87 vs mean 0.52, SD 0.44; mean 0.66, SD 0.52; and mean 0.68, SD 0.43 per 1000 children; *P*<.001). Jurisdictions with stronger reporting requirements, broader definitions, and greater use of alternative response systems (cluster 1) had the lowest incidence and recurrence. No statistically significant differences in maltreatment fatalities were observed across clusters (*P*=.89). However, in a subanalysis of 18 fatality-related policy items, clusters differed significantly in fatality rates, with jurisdictions characterized by less clearly defined fatality policies exhibiting higher fatality rates (mean 47.91, SD 8.35 vs mean 22.64, SD 11.82; mean 25.63, SD 18.62; and mean 19.03, SD 11.64 per 1,000,000 children; *P*=.03).

**Conclusions:**

State child maltreatment policies form distinct, multidimensional patterns associated with maltreatment outcomes. The findings highlight the importance of evaluating integrated policy frameworks rather than isolated components and demonstrate the use of NLP for large-scale, data-driven policy analysis.

## Introduction

Child maltreatment, which includes both child abuse and neglect, is a critical public health concern in the United States. In fiscal year 2023, the most recent year for which information is available, there were 546,159 reported children who experienced child maltreatment, with an estimated 2000 children dying from maltreatment [1]. Child maltreatment is associated with an increased risk of adverse health outcomes and risk behaviors, including substance use, mental health disorders, chronic physical conditions, and premature mortality [2-5]. The economic burden is substantial, with the total lifetime cost estimated at US $2.94 trillion for investigated cases and US $563 billion for substantiated cases in the federal fiscal year 2018 [6].

Child maltreatment policy refers to states’ definitions of child abuse and neglect, along with related regulations that shape child welfare practices. These policies determine how allegations are referred, reported, investigated, and managed [7]. For example, mandated reporting expansion refers to policies that broaden the categories of individuals required to report suspected maltreatment (eg, extending beyond professionals such as teachers and health care providers to include additional occupational groups or, in some states, all adults) [8]. Similarly, policies such as centralized intake systems standardize how reports are received and screened, while differential response systems allow for alternative, noninvestigative pathways for lower-risk cases [9]. Together, these policy components influence both the volume and characteristics of cases that enter and progress through the child welfare system.

At the federal level, the Child Abuse Prevention and Treatment Act (CAPTA), first enacted in 1974 and reauthorized multiple times since, establishes minimum standards for state definitions of abuse and neglect and conditions federal funding on states’ compliance with these requirements [10]. However, states retain substantial discretion in translating these federal requirements into policy and practice, resulting in significant cross-state variation [7]. This variation leads to substantial differences in reported maltreatment rates and intervention effectiveness, with substantiation rates ranging from 5% to 45% across states [11].

Research suggests that states spending an additional US $1000 per person in poverty on benefits such as cash aid, child care, or Medicaid assistance saw 4.3% fewer maltreatment reports and 4.0% fewer substantiations [12]. Additionally, states that implemented mandated reporting expansion, centralized intake, and increased state staffing saw a 32% increase in maltreatment reports, although substantiated reports declined by 5% to 6% [7]. In contrast, states adopting both differential response and higher standards of proof experienced a 24% decrease in substantiated reports, with differential response accounting for 11% and higher proof standards contributing between 12% and 13% [7]. A separate national quasi-experimental study using data from 2004 to 2017 found that states with differential response programs had approximately 19% fewer substantiated reports and a 17% reduction in foster care use relative to states without differential response [9]. These literatures reflect the complex and sometimes countervailing ways in which policy components interact with one another and with local implementation contexts.

However, existing studies have largely examined individual policies or small sets of policy features in isolation. Less is known about how multiple policy components co-occur within states to form broader policy environments, or how states differ systematically across key policy domains. This limitation is important because child welfare systems operate as integrated structures, where policies governing reporting, screening, investigation, and service response interact with one another. As a result, the relationship between any single policy component and observed outcomes may depend on the broader policy context in which it is embedded.

Recent advancements in natural language processing (NLP), particularly the development of large language models (LLMs), have begun to transform policy research and child welfare research. In policy text mining, zero-shot and few-shot classification have been applied to large corpora of legislative text, demonstrating scalability without extensive hand-labeled training data [13]. Other work has used NLP to construct interpretable, theory-driven measures of legislative quality from statutory text [14] and to track policy status across large sets of climate and environmental documents [15-17]. In child welfare research, NLP and related methods have focused on case-level rather than policy-level data. Predictive risk modeling using structured administrative records has estimated individual maltreatment risk, in some cases outperforming existing screening tools [18,19], while NLP applied to case notes and health records has identified instances of maltreatment [20-23]. These efforts enhanced data-driven decision-making within child welfare agencies by allowing for more nuanced insights into family dynamics and case histories [24]. These approaches demonstrate NLP’s value for surfacing information from unstructured text, but case note narratives have also been shown to embed caseworker bias and uncertainty, raising caution about their use in downstream predictive tasks [25]. However, none of this literature has applied NLP to the formal statutory and regulatory policy documents that define state child welfare systems, as distinct from case-level clinical or casework text.

This study uses NLP to identify key components of state child maltreatment policies, examine how states differ across these components, and assess how these policy configurations are associated with maltreatment outcomes in the United States. By characterizing the multidimensional structure of state policy environments, this study aims to provide a more comprehensive understanding of how policy variation corresponds to differences in observed child welfare outcomes.

## Methods

### Overview

This study was reported in accordance with the STROBE (Strengthening the Reporting of Observational Studies in Epidemiology) guidelines. The overall analysis proceeded through 3 stages, as illustrated in Figure 1: comparing and evaluating 6 NLP models and quantifying the 411 state policy items (“Quantification of State Maltreatment Policy Using NLP” section), identifying latent policy dimensions via factor analysis and jurisdiction grouping via k-means clustering (“Exploratory Factor Analysis” and “Clustering Analysis” sections), and comparison of demographic, economic, and maltreatment outcomes across the resulting policy clusters (“Demographic, Economic, and Outcome Comparisons” section).

**Figure 1:**
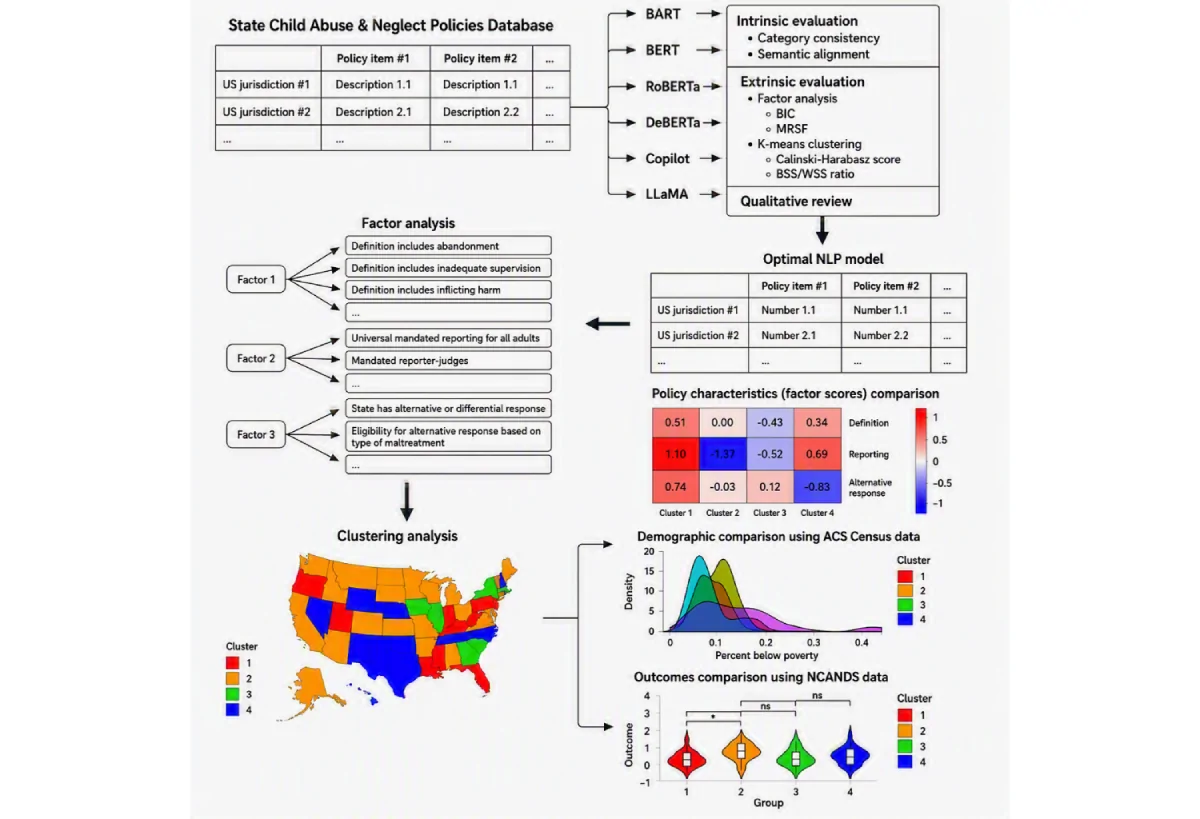
Analytical workflow for evaluating state child maltreatment policy configurations using natural language processing (NLP), exploratory factor analysis, and clustering among 52 US jurisdictions in 2021. ACS: American Community Survey; BART: bidirectional and auto-regressive transformer; BERT: bidirectional encoder representations from transformers; BIC: Bayesian information criterion; BSS/WSS: between-cluster sum of squares to within-cluster sum of squares ratio; DeBERTa: decoding-enhanced BERT with disentangled attention; MRSF: multiple R-squared of scores with factors; NCANDS: National Child Abuse and Neglect Data System; RoBERTa: robustly optimized BERT approach.

### Data Sources

This study used data from 3 sources. State-level child abuse and neglect policy data from 2021 were obtained from the State Child Abuse & Neglect Policies Database (SCAN), which documents state child welfare laws and policies across all 50 US states, the District of Columbia, and Puerto Rico. The database contains 411 standardized policy variables covering state-specific definitions of child abuse and neglect, reporting policies, screening policies, investigation policies, child welfare responses, child welfare system context, and child fatality–related policies in calendar year 2021 [26]. SCAN captures written state laws and policies from publicly available or state-provided sources, including state statutes, regulations, and agency policy documents. Policy variables are coded using a standardized protocol and are verified by state agency contacts when feasible.

Additionally, child welfare agency– and child-level maltreatment data from 2021 were sourced from the National Child Abuse and Neglect Data System (NCANDS), a federally sponsored program administered by the US Department of Health and Human Services [27,28]. NCANDS collects and analyzes state-reported data on child abuse and neglect known to child protective services, as mandated by the CAPTA [27,28]. Child-level records were used to derive state-level measures of maltreatment incidence and recurrence by aggregating individual cases. Maltreatment fatality rates were obtained from the NCANDS agency-level data because some fatality information is not available in the child-level files for confidentiality and data security reasons.

Finally, demographic and economic data from 2021 were drawn from the American Community Survey, an annual survey conducted by the US Census Bureau that provides detailed estimates of social, economic, housing, and demographic characteristics at the state and local levels [29].

### Study Cohort and Outcomes

The study cohort included all 50 states in the United States, the District of Columbia, and the Commonwealth of Puerto Rico, with 411 state maltreatment policy items assessed in each jurisdiction in calendar year 2021. Each policy item represents a specific child welfare policy provision (eg, a mandated reporting requirement, definition of child maltreatment, screening procedure, or investigation practice) and is organized within the SCAN database into predefined policy domains, including child maltreatment definitions, mandated reporting, screening, investigations, child welfare responses, and child welfare system context (Table S1 in Multimedia Appendix 1). These domains are part of the original SCAN database structure. Policy items vary in measurement format depending on the underlying policy provision and include binary, categorical, ordinal, and descriptive variables. Although the SCAN database organizes policy items into predefined domains, these domains were used only to describe the source variables. The subsequent analyses identified latent policy dimensions empirically using exploratory factor analysis (EFA) rather than relying on the original SCAN categorization.

Maltreatment outcomes were evaluated for individuals aged <18 years. While some states, such as Illinois, allow youth aged 18 years or older to remain in care, others do not. To ensure consistent denominators across jurisdictions, individuals aged 18 years or older were excluded.

The study examined three outcomes: (1) incidence of maltreatment, measured as the number of substantiated or indicated maltreatment cases per 1000 children aged <18 years in each state; (2) maltreatment recurrence, defined as the number of children previously identified as having experienced maltreatment who experienced subsequent incidents per 1000 children in the population; and (3) maltreatment fatalities, defined as the number of deaths resulting from maltreatment per 1,000,000 children. For the first 2 outcomes, only substantiated or indicated maltreatment recorded by state child protective services in NCANDS with report dates in 2021 were included. Although these outcomes represent related dimensions of child maltreatment burden, they capture distinct aspects of child welfare involvement and were analyzed separately rather than modeled as a combined outcome.

### Quantification of State Maltreatment Policy Using NLP

#### Overview

The primary study variable was state maltreatment policy. To prepare these data, 411 state maltreatment policy items from 2021 across 52 US jurisdictions were processed using NLP models.

#### NLP Approach

This study applied NLP methods to classify and analyze 411 state maltreatment policy items from 2021 across 52 US jurisdictions. A zero-shot classification [30], which uses pretrained models to assign labels without task-specific fine-tuning, was selected because the policy data were not labeled for the specific classification tasks required in this study. Alternative approaches, such as supervised or few-shot classification, typically require curated training examples for each category, which were not available and would require substantial manual annotation. Zero-shot methods provide a scalable and consistent framework for applying predefined policy categories across heterogeneous statutory text while avoiding potential bias introduced through hand-labeled training data.

To evaluate the robustness of this approach, 6 NLP models were compared, representing distinct architectures and training paradigms, including encoder-based, sequence-to-sequence, and generative LLMs. Comparing these models allows assessment of whether classification results are consistent across fundamentally different modeling approaches, and whether more recent generative models provide advantages over traditional transformer-based classifiers in capturing the structure of policy text. The 6 models evaluated were as follows:

1. Bidirectional and auto-regressive transformer (BART) [31], a sequence-to-sequence transformer with a bidirectional encoder and an autoregressive decoder, designed for classification and text generation tasks;
2. Bidirectional encoder representations from transformers (BERT) [32], a bidirectional transformer encoder trained using masked-language modeling for deep contextual text understanding;
3. Robustly optimized BERT approach (RoBERTa) [33], an optimized version of BERT trained longer and on larger corpora to improve robustness and performance;
4. Decoding-enhanced BERT with disentangled attention (DeBERTa) [34], a transformer model that enhances BERT/RoBERTa with disentangled attention and improved position encoding;
5. Microsoft Copilot [35], generative transformer models adapted for instruction-following and classification;
6. Meta LLaMA 3.1 [36], an LLM in the LLaMA family, trained as an autoregressive transformer with strong general-purpose language understanding capabilities.

The models evaluated each policy item independently by assessing whether each jurisdiction’s policy text supports or contradicts the policy item’s title description. For example, for the policy item titled “How is reporting decentralized?,” jurisdiction responses include “Each county has its own reporting hotline,” “Some counties have their own reporting hotlines,” and “No county has its own reporting hotline.” Each NLP model assigned a likelihood score from 0 to 1 to each response, indicating the degree to which it supported the policy title, where 1 represented the strongest support and 0 the weakest. For instance, the BART model assigned scores of 0.90, 0.81, and 0.59, respectively.

For LLMs, Microsoft Copilot and Meta LLaMA 3.1, the scores were generated by a structured prompt, which was applied separately to each policy item and took the following format:

“Please estimate the likelihood (on a scale from 0 to 1) that the response [A], [B], [C] supports or opposes the statement [Policy Title]. Stronger support should be closer to 1, a neutral or irrelevant stance should be around 0.5, and complete opposition should be close to 0. Provide likelihoods in this format: [A]: score1; [B]: score2; [C]: score3.”

#### NLP Evaluation Metrics

The 6 models were evaluated using intrinsic and extrinsic metrics [37,38]. Intrinsic evaluation measured internal consistency and agreement by testing how well LLM embedding vectors capture the underlying characteristics of the texts, while extrinsic evaluation assessed the performance of key embedding features extracted from the policy documents based on their performance in downstream tasks and analyses [37]. Since later sections of the study applied EFA [39] and k-means clustering [40] to policy data, these techniques were used here as extrinsic evaluation metrics to determine how well different NLP models capture meaningful variation in policy content for the same set of features.

For intrinsic evaluation, 3 metrics were used. First, category consistency was assessed based on the relative ordering of classification scores across response categories. Specifically, items labeled as “Yes” were expected to receive higher scores than those labeled “No,” while intermediate responses such as “Not Applicable,” “Unknown,” or “Missing” were expected to fall between “Yes” and “No.” Second, for descriptive responses, semantic alignment was measured using 2 metrics. Because a true gold standard was unavailable for these responses, 2 proxy gold standards were used: (1) cosine similarity scores, where higher values indicate better semantic alignment, and (2) the average score across all methods. The correlation between each model’s results and these proxy standards, where a higher correlation indicates better agreement, served as the metric to assess semantic alignment.

Extrinsic evaluation assessed EFA and clustering results derived from each NLP model-specific policy representation. In this stage, each model produced a complete set of scores for the 411 policy items across jurisdictions, which served as inputs for EFA and k-means clustering. Model performance was then assessed by comparing goodness-of-fit and clustering metrics to identify which NLP approaches most effectively capture meaningful structure in the policy data. EFA results were evaluated using the Bayesian information criterion (BIC) [41], where lower values indicate better model fit accounting for complexity, and the multiple R-squared of scores with factors (MRSF) [39], where higher values reflect better explanatory power of the factor scores. To explore different latent structures, EFA models with 2, 3, and 4 factors were examined. Clustering performance was analyzed using the Calinski-Harabasz score (CH) [42], where higher values indicate better cluster separation relative to complexity, and the between-cluster sum of squares to within-cluster sum of squares (BSS/WSS) ratio [43], where higher ratios represent more compact and well-separated clusters. Models with 2, 3, and 4 clusters were examined to evaluate clustering performance.

#### Qualitative Review

Finally, a qualitative review explored why certain methods perform better on specific metrics by analyzing model outputs, identifying recurring error patterns, and assessing consistency across similar policy items. This review considered factors such as sensitivity to nuanced language, handling of ambiguous or incomplete responses, and stability in scoring across different policy categories. This review aimed to gain an intuitive understanding of model behavior and to verify that the results aligned with expectations.

Overall model performance was determined by considering results across all intrinsic and extrinsic evaluation metrics rather than relying on a single metric or composite score. No weighting scheme was applied. Instead, models were compared based on their relative performance patterns across evaluation dimensions, with qualitative review used to further examine model behavior, identify potential sources of variation, and assess whether quantitative findings were consistent with expected policy interpretation.

### Covariates

Demographic and economic characteristics were included as covariates to contextualize jurisdiction-level differences. Demographic covariates included age distribution, racial and ethnic composition, and gender. Economic conditions were measured by the proportion of the population living below 50%, 100%, 125%, and 400% of the federal poverty level, capturing varying levels of economic vulnerability.

### Statistical Analysis

#### Exploratory Factor Analysis

Although the SCAN database organizes policy items into predefined domains for documentation purposes, these domains do not necessarily represent latent policy constructs. EFA was conducted on the 411 state maltreatment policy items to identify latent dimensions [39]. Consequently, policy items originating from the same SCAN domain were not constrained to load on the same factor; likewise, policy items from different SCAN domains could load on the same factor if they reflected similar underlying policy characteristics. The number of factors was determined using a scree plot, a graphical tool that helps identify the point where the rate of decrease in explained variance slows [44]. While this plot provides a helpful visual cue, identifying the “elbow” can be subjective and challenging [44,45]. In cases where the exact location of the “elbow” is ambiguous and several factor number choices were similarly justifiable, we opted for the smaller number of factors to ensure concise and easier interpretation.

To identify the underlying themes for each factor, policy items with high factor loadings were reviewed. These factors were then labeled based on the substantive patterns they capture. For each factor in each jurisdiction, factor scores were computed to quantify the degree to which each jurisdiction aligns with the identified policy dimensions [46]. The factor scores provided a reduced representation of the original 411 policy items and were used in subsequent analyses to characterize jurisdiction-level policy configurations.

The internal consistency of policy items contributing to each identified factor was assessed using McDonald ω [47]. ω was calculated based on policy items with absolute factor loadings above 0.4.

#### Clustering Analysis

K-means clustering was applied to the 411 state maltreatment policy items to identify groups of jurisdictions that share similar policy characteristics [40]. The optimal number of clusters was determined using the Silhouette method [48], which evaluated cluster cohesion and separation, along with interpretability considerations, to ensure the resulting groupings were meaningful and practically relevant. After the number of clusters is selected, each jurisdiction is assigned to a cluster based on the similarity of its policy item representations to the identified cluster profiles. To visually illustrate the geographic distribution of policy clusters, a US map is generated, with jurisdictions color-coded according to their assigned cluster. The policy characteristics of each cluster are identified by comparing average factor scores derived from the factor analysis.

Although k-means clustering and factor analysis are distinct techniques, they serve complementary purposes in this study. Factor analysis reduces the dimensionality of the policy items and identifies underlying policy domains, whereas clustering groups jurisdictions based on similarity across the full set of policy features. By conducting clustering on all items and using factor scores for interpretation, this approach preserves the full information available for grouping while enabling a more interpretable, lower-dimensional characterization of cluster differences. Consistency between cluster profiles and factor structures further supports the coherence and robustness of the observed policy patterns.

#### Demographic, Economic, and Outcome Comparisons

To characterize each cluster, policy factor scores were compared across clusters. Then, demographic, economic, and outcome differences were examined across jurisdiction clusters. Statistical differences across clusters were tested using ANOVA for continuous variables [49] and chi-square tests for categorical variables [50]. When significant differences are detected, post hoc pairwise comparisons are conducted to determine which clusters differ from each other. Given the small sample size of 52 US jurisdictions, a significance level of 0.10 is used to reduce the risk of type II errors and detect potential associations that may warrant further investigation [51,52].

### Sensitivity Analyses

#### Leave-One-State-Out Sensitivity Analysis

Cluster robustness was assessed using a leave-one-jurisdiction-out sensitivity analysis. K-means clustering was repeated after sequentially removing each jurisdiction, and agreement between the resulting cluster assignments and the original clustering was evaluated using the adjusted Rand index (ARI) [53]. This analysis was conducted to evaluate whether the identified policy configurations were robust to the exclusion of individual jurisdictions and not driven by any single state’s policy profile.

#### Sensitivity Analysis Including All NCANDS Maltreatment Records

To assess whether findings were sensitive to the definition of maltreatment outcomes, a sensitivity analysis was conducted using all screened-in maltreatment records reported in NCANDS in 2021, regardless of substantiation or indication status. The same outcome comparison procedures across policy clusters were applied to assess whether the observed associations between policy configurations and maltreatment outcomes remained consistent under an alternative maltreatment definition.

### Subanalysis

To further examine policies specifically related to maltreatment fatalities, a separate and independent clustering analysis was conducted using only the 18 fatality-related policy items (Table S2 in Multimedia Appendix 1). Unlike the primary analysis, which clustered jurisdictions based on all 411 policy items, this subanalysis clustered jurisdictions solely according to fatality-related policies.

K-means clustering is applied to these 18 policy items to group jurisdictions with similar fatality-related policy characteristics. The maltreatment fatality rates across these clusters are then compared to identify any notable differences related to policy configurations. A violin plot—a visualization that combines a box plot with a density curve—is used to display the distribution of fatality rates by cluster. Statistical tests are conducted to determine whether the observed differences in fatality rates are statistically significant.

### Ethical Considerations

This study was approved by the Northwestern University Institutional Review Board (STU00222024). The study consisted of a secondary analysis of deidentified administrative data obtained from the National Data Archive on Child Abuse and Neglect (NDACAN). Data access was granted by NDACAN under its Data Use Agreement, and all analyses were conducted in accordance with the terms of the Data Use Agreement and applicable institutional policies. Because the study involved retrospective analysis of existing deidentified data with no direct participant contact, the Northwestern University Institutional Review Board waived the requirement for informed consent.

The authors did not have access to information that could directly or indirectly identify individual participants during or after data collection. All datasets provided by NDACAN were deidentified prior to release, and analyses were conducted using secure data management procedures in compliance with the NDACAN Data Use Agreement, which prohibits attempts to reidentify participants.

No participants were recruited for this secondary data analysis; therefore, no compensation was provided.

## Results

### Study Cohort Characteristics

The study included 52 jurisdictions in the United States: all 50 states, the District of Columbia, and the Commonwealth of Puerto Rico. In calendar year 2021, these jurisdictions had a combined population of 72,838,819 individuals aged <18 years, with 18,416,713 (25.3%) aged <5 years, and 54,422,106 (74.7%) between ages 5 and 17 years. In the same year, there were 3,774,528 maltreatment records, each representing a unique report and child combination, involving 3,134,446 unique children. Of these, 751,283 records were indicated, affecting 692,891 unique children. Although NCANDS contains individual-level child welfare records, the unit of analysis for this study was the jurisdiction. Individual-level records were aggregated to the jurisdiction level to derive maltreatment outcomes.

### NLP Results

Table 1 presents the comparison of the 6 NLP models: BART, BERT, RoBERTa, DeBERTa, Copilot, and LLaMA 3.1. For intrinsic evaluation, category consistency scores indicate that DeBERTa and Copilot achieve the best performance (0.95). Semantic alignment results vary across metrics. Based on the similarity metric, LLaMA 3.1 performs best (0.21), followed by BART (0.19) and DeBERTa (0.14). In contrast, when evaluated using the average score metric, Copilot ranks highest (0.91).

For extrinsic evaluation, RoBERTa demonstrates the best performance in factor analysis across all models, achieving the lowest BIC values and the highest MRSF scores for different numbers of factors (2-factor: BIC=–601336; MRSF=0.98; 3-factor: BIC=–561327; MRSF=0.98; 4-factor: BIC=–535315; MRSF=0.98). DeBERTa follows closely with competitive results (2-factor: BIC=–581536; MRSF=0.98; 3-factor: BIC=–550143; MRSF=0.98; 4-factor: BIC=–526241; MRSF=0.97). For clustering, DeBERTa outperforms other models, achieving the highest CH and BSS/WSS ratios across all cluster solutions (2 clusters: CH=4.45; BSS/WSS=1.07; 3 clusters: CH=3.90; BSS/WSS=1.08; 4 clusters: CH=3.28; BSS/WSS=1.09).

The qualitative review showed key sources of error and model-specific challenges (Table S3 in Multimedia Appendix 1). For category consistency, RoBERTa had the lowest score due to struggles with negations (eg, “does not” or “failure to report”), leading to misclassifications, while LLaMA 3.1 often hallucinated artificial logic. In semantic alignment, LLM performance varied—while these models sometimes fabricated justifications instead of following response structures, they also demonstrated a better understanding of longer descriptive texts. However, evaluating semantic alignment was challenging, as similarity and average scores served as imperfect proxies. When comparing similarity and average score approaches, DeBERTa was neither the best nor the worst, performing well in some contexts but falling short in others. Finally, in extrinsic evaluation, DeBERTa outperformed other models, likely because it better preserves structural relationships and maintains meaningful variance in the data.

Based on intrinsic, extrinsic, and qualitative evaluations, DeBERTa was the best-performing model overall, achieving the highest category consistency, strong semantic alignment, the second-best factor analysis performance, and the best clustering result. Because a gold-standard annotation of policy items did not exist, a formal error rate was not calculated.

**Table 1:** Comparison of 6 natural language processing methods for quantifying state child maltreatment policies across 52 US jurisdictions using 2021 State Child Abuse & Neglect Policies Database data.

			BART^a^		BERT^b^		RoBERTa^c^		DeBERTa^d^		Copilot		LLaMA
Intrinsic													
	Category consistency^e^	0.90		0.81		0.73		0.95		0.95		0.78	
	Semantic alignment—similarity^f^	0.19		0.04		0.10		0.14		0.10		0.21	
	Semantic alignment—average^g^	0.77		0.80		0.35		0.68		0.91		0.71	
Extrinsic: factor analysis													
	Factor analysis 2, BIC^h,i^	–464,493		–460,680		–601,336		–581,536		–457,318		–442,535	
	Factor analysis 2, MRSF^i^	0.97		0.97		0.98		0.98		0.96		0.97	
	Factor analysis 3, BIC^i^	–439,225		–439,606		–561,327		–550,143		–437,013		–425,035	
	Factor analysis 3, MRSF^i^	0.97		0.97		0.98		0.98		0.96		0.97	
	Factor analysis 4, BIC^i^	–420,815		–423,379		–535,315		–526,241		–419,011		–409,666	
	Factor analysis 4, MRSF^i^	0.97		0.97		0.98		0.97		0.96		0.96	
Extrinsic: k-means													
	K-means 2, CH^i^	3.57		2.81		2.67		4.45		2.69		2.81	
	K-means 2, BSS/WSS^i^	1.06		1.04		1.04		1.07		1.03		1.03	
	K-means 3, CH^i^	3.07		2.06		2.76		3.90		2.11		2.65	
	K-means 3, BSS/WSS^i^	1.07		1.03		1.05		1.08		1.03		1.05	
	K-means 4, CH^i^	2.80		2.49		2.74		3.28		2.04		2.79	
	K-means 4, BSS/WSS^i^	1.07		1.06		1.07		1.09		1.04		1.07	

^a^BART: bidirectional and auto-regressive transformer.

^b^BERT: bidirectional encoder representations from transformers.

^c^RoBERTa: robustly optimized BERT approach.

^d^DeBERTa: decoding-enhanced BERT with disentangled attention.

^e^Category consistency: evaluates whether categorical descriptions align with expected patterns. Higher values indicate better consistency.

^f^Semantic alignment—similarity: using a similarity score as the proxy gold standard to evaluate semantic alignment. Higher values indicate better semantic alignment.

^g^Semantic alignment—average: using the average score across all methods as the proxy gold standard to evaluate semantic alignment. Higher values indicate better semantic alignment.

^h^BIC: Bayesian information criterion.

^i^Extrinsic evaluation metric row titles follow the format “[Method] [Number], [Metric],” where the number represents either the number of factors in exploratory factor analysis or the number of clusters in k-means clustering. The metrics include BIC, multiple R-squared of scores with factors (MRSF), Calinski-Harabasz score (CH), and the between-cluster sum of squares to within-cluster sum of squares ratio (BSS/WSS). Lower BIC values indicate a better fit, while higher values are preferable for MRSF, CH, and BSS/WSS.

### EFA Results

The scree plot (Figure S1 in Multimedia Appendix 1) showed that although a clear “elbow” did not appear until over 50 factors—an impractically large number for interpretation—the decline in eigenvalues slowed around 3 factors, suggesting a point of diminishing returns. We interpreted this inflection as a minor elbow and selected a 3-factor solution to avoid the complexity of higher-dimensional models while still retaining meaningful structure.

The items with the highest absolute factor loadings for each factor (Table S4 in Multimedia Appendix 1) indicated that Factor 1 represented the definition domain, Factor 2 focused on the reporting domain, and Factor 3 captured the alternative response domain. Factor 1 (Definition) reflected the scope of maltreatment definitions, with higher scores corresponding to broader definitions that explicitly included emotional harm, abandonment, and sexual abuse, as well as more stringent safe haven requirements. Factor 2 (Reporting) covered policies related to mandated reporting, where higher scores were associated with a greater number of mandated reporters, more stringent penalties for failure to report, and a reduced emphasis on reporter training. Factor 3 (Alternative Response) captured states’ implementation of differential response systems, with higher scores indicating broader eligibility for alternative responses based on case characteristics such as risk level and maltreatment type.

The extracted policy dimensions demonstrated high internal consistency. McDonald ω values were 0.996, 0.959, and 0.938 for the 3 factors, indicating strong coherence among policy items contributing to each latent policy dimension.

### Clustering Results

The k-means clustering analysis was used to identify distinct clusters based on policy characteristics. The optimal number of clusters was determined using the silhouette width, which peaked at 2 and 4 clusters (Figure S2 in Multimedia Appendix 1). To better capture the diversity and variability in the policy characteristics across jurisdictions, 4 clusters were chosen.

A total of 4 distinct policy clusters with varying geographic distributions across US states were identified (Figure 2). Cluster 1 (n=12) was concentrated in the South and parts of the West, including Texas, Oklahoma, and several Rocky Mountain states. Cluster 2 (n=6) was scattered, appearing in parts of the central, Northeast, and Southeast. Cluster 3 (n=23), the largest cluster, covered much of the central and western regions, as well as parts of the northern region, including the Midwest, Great Plains, and Mountain West. Cluster 4 (n=11) was primarily located in the southern and southeastern US, with a few states in the West also assigned to this group.

The policy characteristics of each cluster were assessed using factor scores (Table 2 and Figure S3 in Multimedia Appendix 1). Cluster 1 had the highest scores across all 3 domains (Definition: 0.51, Reporting: 1.05, Alternative Response: 0.74), reflecting broad maltreatment definitions, strict mandated reporting policies with strong penalties, and extensive use of differential response systems. Cluster 2 showed the lowest Reporting score (–1.37), indicating fewer mandated reporting requirements and weaker penalties. Cluster 3 had the lowest Definition score (–0.43), suggesting narrower maltreatment definitions and more lenient safe haven policies. Cluster 4 had the lowest Alternative Response score (–1.05), reflecting the limited implementation of differential response systems and a likely greater reliance on traditional case-handling approaches.

Regarding demographic and economic differences (Table 3), there were no significant differences in child population among clusters (cluster 1: 1.2 million; cluster 2: 2.1 million; cluster 3: 1.3 million; cluster 4: 1.4 million; *P*=.76). Demographically, there were minimal differences in age, gender, and race/ethnicity distributions across clusters. However, poverty status showed significant differences (Figure S4 in Multimedia Appendix 1), with cluster 4 showing a higher proportion of children below poverty levels (cluster 1: 0.17; cluster 2: 0.16; cluster 3: 0.15; cluster 4: 0.21; *P*=.048).

Regarding maltreatment outcomes (Table 4), cluster 2 exhibited the highest incidence of maltreatment, while cluster 1 had the lowest (cluster 1: mean 9.65, SD 4.10; cluster 2: mean 17.51, SD 6.13; cluster 3: mean 10.28, SD 6.47; cluster 4: mean 11.74, SD 6.47; *P*=.07). A similar trend was observed for maltreatment recurrence, with cluster 2 showing the highest rate (cluster 1: mean 0.52, SD 0.44; cluster 2: mean 1.74, SD 0.87; cluster 3: mean 0.66, SD 0.52; cluster 4: mean 0.68, SD 0.43; *P*<.001). In contrast, there were no significant differences in maltreatment fatalities across clusters (cluster 1: mean 21.63, SD 14.83; cluster 2: mean 25.74, SD 14.70; cluster 3: mean 25.80, SD 15.64; cluster 4: mean 23.20, SD 18.80; *P*=.89). Pairwise comparisons (Figure 3) revealed that cluster 2 had a significantly higher incidence of maltreatment than Clusters 1 and 4 and a significantly higher recurrence of maltreatment than Clusters 1, 3, and 4.

**Figure 2:**
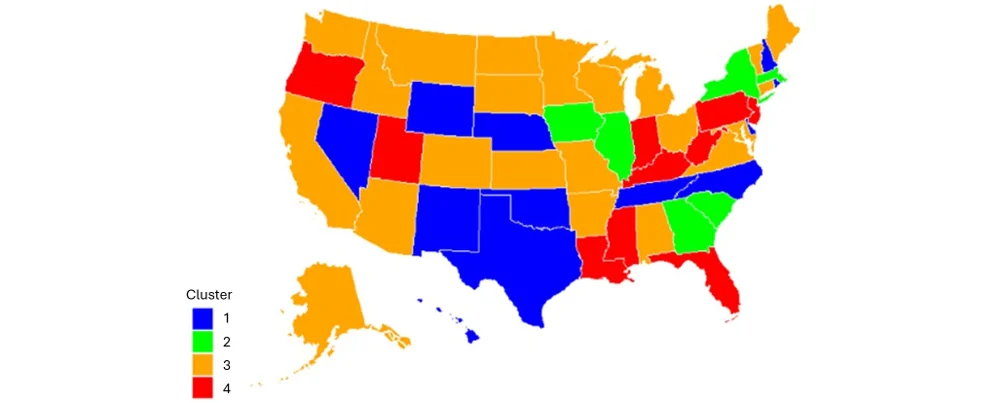
Geographic distribution of state child maltreatment policy clusters across 52 US jurisdictions based on 2021 State Child Abuse & Neglect Policies Database data.

**Table 2:** Comparison of latent child maltreatment policy factor scores across jurisdiction clusters identified through 2021 policy data from the State Child Abuse & Neglect Policies Database.

	Cluster 1 (n=12), mean (SD)	Cluster 2 (n=6), mean (SD)	Cluster 3 (n=23), mean (SD)	Cluster 4 (n=11), mean (SD)	*P* value^a^
Definition	0.51 (1.19)	0.00 (1.09)	–0.43 (0.58)	0.34 (1.15)	.03
Reporting	1.05 (0.42)	–1.37 (0.37)	–0.53 (0.44)	0.69 (0.83)	<.001
Alternative response	0.74 (0.76)	–0.03 (0.79)	0.12 (0.95)	–1.05 (0.23)	<.001

^a^*P* values are based on 1-way ANOVA tests.

**Table 3:** Comparison of demographic characteristics and poverty levels across state child maltreatment policy clusters among 52 US jurisdictions in 2021.

					Cluster 1 (n=12), mean (SD)		Cluster 2 (n=6), mean (SD)		Cluster 3 (n=23), mean (SD)		Cluster 4 (n=11), mean (SD)		*P* value^a^
Child population (in millions)					1.2 (2.0)		2.1 (1.2)		1.3 (1.7)		1.4 (1.1)		.76
Age (years)													
		<5		0.25 (0.01)		0.25 (0.01)		0.26 (0.02)		0.25 (0.02)		.44	
		5-17		0.75 (0.01)		0.75 (0.01)		0.74 (0.02)		0.75 (0.02)		.44	
Gender													
			Male	0.50 (0.01)		0.49 (0.01)		0.50 (0.01)		0.49 (0.01)		.13	
			Female	0.50 (0.01)		0.51 (0.01)		0.50 (0.01)		0.51 (0.01)		.13	
Race and ethnicity													
			White alone	0.62 (0.19)		0.64 (0.12)		0.70 (0.15)		0.67 (0.18)		.56	
			Black alone	0.08 (0.07)		0.15 (0.10)		0.10 (0.11)		0.12 (0.11)		.52	
			Hispanic or Latino	0.18 (0.14)		0.12 (0.06)		0.11 (0.10)		0.19 (0.28)		.46	
Poverty status													
	Below 50% poverty level				0.06 (0.01)		0.06 (0.01)		0.06 (0.01)		0.08 (0.05)		.11
	Below poverty level				0.17 (0.04)		0.16 (0.02)		0.15 (0.03)		0.21 (0.10)		.048
	Below 125% poverty level				0.21 (0.05)		0.20 (0.03)		0.19 (0.03)		0.26 (0.12)		.04
	Below 400% poverty level				0.13 (0.03)		0.13 (0.02)		0.12 (0.02)		0.16 (0.09)		.06

^a^*P* values are based on 1-way ANOVA tests.

**Table 4:** Comparison of child maltreatment outcomes across state child maltreatment policy clusters among 52 US jurisdictions in 2021.

	Cluster 1 (n=12), mean (SD)	Cluster 2 (n=6), mean (SD)	Cluster 3 (n=23), mean (SD)	Cluster 4 (n=11), mean (SD)	*P* value^a^
Incidence of maltreatment	9.65 (4.10)	17.51 (6.13)	10.28 (6.47)	11.74 (6.47)	.07
Maltreatment recurrence	0.52 (0.44)	1.74 (0.87)	0.66 (0.52)	0.68 (0.43)	<.001
Maltreatment fatalities	21.63 (14.83)	25.74 (14.70)	25.80 (15.64)	23.20 (18.80)	.89

^a^*P* values are based on 1-way ANOVA tests.

**Figure 3:**
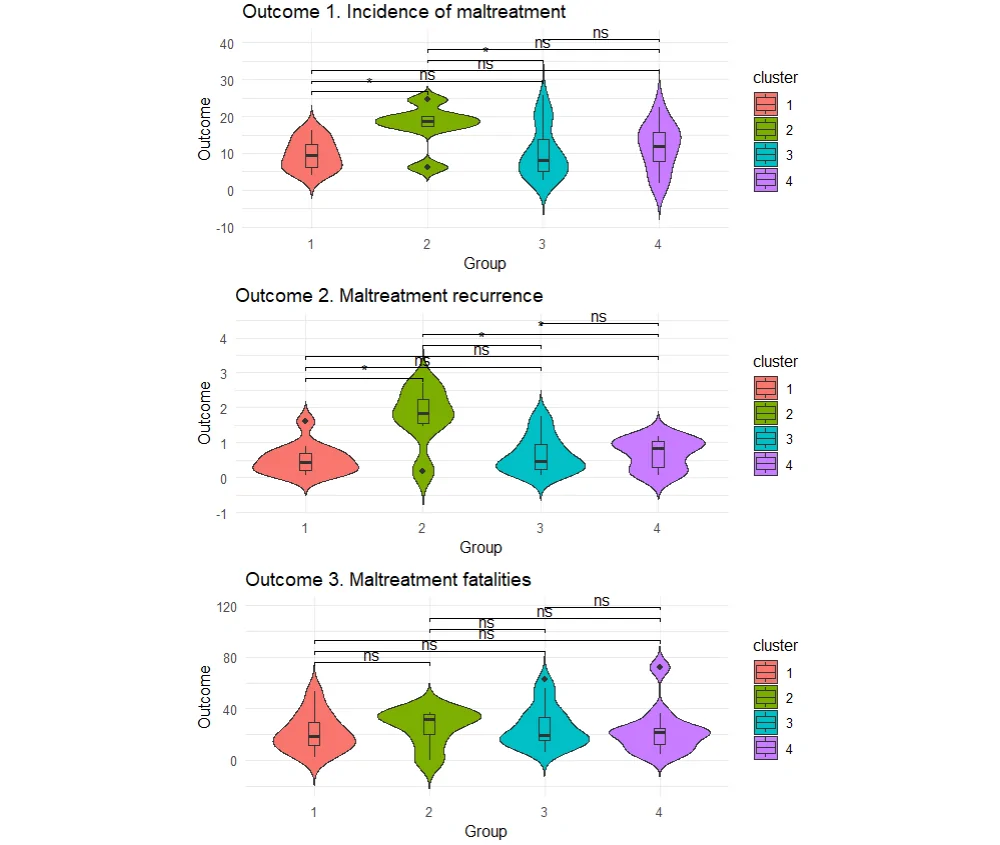
Pairwise comparisons of maltreatment outcomes across state child maltreatment policy clusters among 52 US jurisdictions in 2021.

### Sensitivity Analyses

The leave-one-jurisdiction-out sensitivity analysis demonstrated high cluster stability (mean ARI 0.984, range 0.726-1.000), suggesting that the identified policy configurations were not driven by individual jurisdictions.

A sensitivity analysis using all screened-in NCANDS maltreatment records in 2021, regardless of substantiation or indication status, showed similar findings. Cluster 2 continued to have the highest maltreatment incidence and recurrence, although only the difference between cluster 2 and cluster 3 remained statistically significant (Figure S5 and Table S5 in Multimedia Appendix 1).

### Subanalysis Results

An independent clustering analysis based solely on the 18 fatality-related policy items (Figure S6 in Multimedia Appendix 1) identified 4 fatality-policy clusters, which are distinct from the 4 policy clusters reported in the primary analysis. Among these fatality-policy clusters, cluster 2 had the least clear definition for child fatalities and near-fatalities, with the lowest scores in “child fatalities definition includes injury,” “child fatalities definition includes death of children in foster care,” “child fatalities definition includes other,” “child near-fatalities definition includes general reference to condition/injury,” and “child near-fatalities definition includes specific injury or treatment” (Table S6 in Multimedia Appendix 1).

Regarding outcomes, there were no significant differences among fatality-related policy clusters in terms of incidence of maltreatment or maltreatment recurrence (Table 5). However, a significant difference was found in maltreatment fatalities (cluster 1: mean 22.64, SD 11.82; cluster 2: mean 47.91, SD 8.35; cluster 3: mean 25.63, SD 18.62; cluster 4: mean 19.03, SD 11.64; *P*=.03).

Pairwise comparisons showed that cluster 2 had significantly higher maltreatment fatalities compared to Clusters 1, 3, and 4 (Figure 4). The fatality distribution for cluster 2 was relatively narrow and centered around a higher median, indicating consistently elevated fatality rates. In contrast, cluster 3 showed a wider and more skewed distribution with greater variability, while clusters 1 and 4 showed more moderate and tightly clustered fatality rates with lower medians.

**Table 5:** Comparison of maltreatment outcomes across jurisdictions grouped by fatality-related policy clusters.

	Cluster 1 (n=15), mean (SD)	Cluster 2 (n=3), mean (SD)	Cluster 3 (n=20), mean (SD)	Cluster 4 (n=14), mean (SD)	*P* value^a^
Incidence of maltreatment	10.57 (7.11)	14.56 (6.21)	11.21 (5.63)	11.43 (7.35)	.82
Maltreatment recurrence	0.61 (0.55)	0.89 (0.21)	0.75 (0.57)	0.89 (0.84)	.68
Maltreatment fatalities	22.64 (11.82)	47.91 (8.35)	25.63 (18.62)	19.03 (11.64)	.03

^a^*P* values are based on 1-way ANOVA tests.

**Figure 4:**
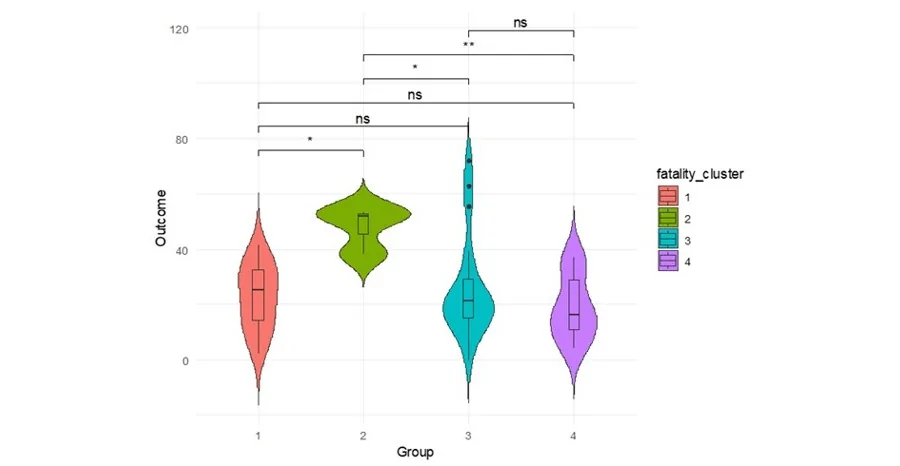
Pairwise outcome differences for subanalysis based on the 18 maltreatment fatality policy items.

## Discussion

### Summary of Findings

This study analyzed state child maltreatment policies and identified distinct policy clusters associated with maltreatment outcomes. Key findings indicated that states with the fewest mandated reporting requirements and weakest penalties (cluster 2) were associated with the highest substantiated or indicated maltreatment incidence and recurrence rates recorded in NCANDS, whereas states with the strongest reporting requirements, broadest maltreatment definitions, and most extensive use of alternative response (cluster 1) exhibited the lowest rates of substantiated or indicated maltreatment and recurrence. Additionally, states with broad maltreatment definitions and stringent reporting policies (cluster 4) had a high proportion of individuals below the poverty level, although maltreatment outcomes in these states were not significantly different.

### Interpretation of Findings

Previous studies found that mandated reporting policies increased the number of maltreatment reports but did not have a significant impact on substantiated or indicated maltreatment incidence [7,54]. This study expanded upon them by suggesting that mandated reporting policies, when coupled with broad definitions of maltreatment and alternative response systems, were associated with lower rates of substantiated or indicated maltreatment and recurrence. These findings highlight the importance of considering the broader policy context in evaluating the role of mandated reporting.

While previous studies had linked child poverty to higher maltreatment reports [55,56], this study found that clusters with broader definitions and limited alternative response systems tended to be more economically disadvantaged. Although maltreatment outcomes in these states were slightly higher, differences were not statistically significant. These findings suggest the importance of considering how policy frameworks may intersect with socioeconomic conditions to shape child welfare involvement.

This relationship between child fatality definitions and maltreatment fatality rates has not been explicitly examined in prior research. These findings suggested that a lack of definitional specificity may influence reporting and intervention efforts, potentially contributing to higher fatality rates.

### Policy Implications

The findings suggested several considerations for child maltreatment policy. First, states may benefit from integrating mandated reporting with coordinated prevention and response approaches. Strengthening reporting requirements while also implementing broad maltreatment definitions and alternative response systems was associated with lower rates of substantiated or indicated maltreatment and recurrence recorded in NCANDS. Developing comprehensive frameworks that balance reporting with both preventive services and supportive response options, such as alternative response systems, could help improve child welfare outcomes [57-59].

Additionally, states with broad maltreatment definitions and stringent reporting policies had higher rates of poverty. While maltreatment outcomes in these states were not significantly different, the overlap between expansive definitions and economic disadvantage raises concerns that reports of maltreatment may, at times, reflect conditions of financial hardship rather than actual harm. This highlights the importance of refining definitions to ensure they do not conflate poverty with neglect [60,61].

Standardizing child fatality definitions across jurisdictions could also enhance the accuracy of reporting and effectiveness of intervention efforts. Establishing clear and consistent criteria for classifying and reporting child fatalities related to maltreatment may improve cross-state comparisons and inform more effective prevention strategies [62,63].

Lastly, the use of NLP and other advanced analytics in policy analysis demonstrated the potential for data-driven approaches in shaping more effective child welfare policies. Leveraging these technologies could provide valuable insights into policy development and evaluation.

### Limitations and Future Directions

This study had several limitations. First, while the NLP models quantified policy characteristics, they may struggle with nuanced interpretations, particularly in complex policy language. Second, the study relied on state-reported NCANDS data, which may contain inconsistencies in reporting practices across jurisdictions. Third, the analysis was limited to policies in 2021, as more recent data were not available at the time of the study, potentially missing subsequent policy changes. Fourth, the cross-sectional design limited the ability to assess causal relationships between policy configurations and maltreatment outcomes. Finally, semantic alignment was challenging to evaluate due to the absence of a true gold standard, and reliance on proxy measures may not fully capture model accuracy.

Beyond these limitations, several sources of structural uncertainty are worth noting, as their exact magnitude remains largely unknown. Cross-state variation in reporting practices means observed outcome differences may partly reflect how jurisdictions define and record cases rather than underlying policy effects alone. Lag times between policy enactment and implementation add further uncertainty, since coded 2021 policies may not yet have been fully operationalized, while outcomes that year may partly reflect earlier policy environments. Tracking inconsistencies across state data systems also introduces measurement noise that is difficult to quantify but may affect the stability of cluster-outcome associations.

Future research should refine NLP approaches to enhance policy interpretation, explore longitudinal analyses to assess policy changes over time, and examine the impact of federal initiatives such as the Family First Prevention Services Act on state policy configurations and child welfare outcomes. With larger longitudinal datasets, future studies could also explore integrated machine learning or hierarchical modeling approaches to jointly model co-occurring policy configurations and multiple child welfare outcomes while accounting for temporal relationships and potential confounding factors. Recent advances in causal machine learning methods for policy evaluation [64] could further help address confounding in future work assessing the causal impact of specific policy configurations on maltreatment outcomes. Finally, parametric approaches integrating multiple co-occurring policies to predict population-level outcomes have also been developed in other domains, such as epidemic modeling of nonpharmacological interventions [65], and may offer a useful direction for future longitudinal extensions of this work.

### Conclusions

This study identified distinct state-level child maltreatment policy configurations and examined their associations with maltreatment outcomes using an NLP-based analytical framework. This study advanced NLP-based analysis of child maltreatment policies across all US states, offering a scalable, reproducible approach to identify policy patterns. By integrating EFA and clustering, this study moved beyond descriptive comparisons and uncovered latent policy structures and their associations with demographic, economic, and child maltreatment outcomes. These findings offered a data-driven basis for comparing policies, identifying gaps, and informing reform.

## Appendix

Multimedia Appendix 1 Supplementary figures and tables providing factor analysis diagnostics, cluster determinations, geographic distributions, outcome comparisons, and SCAN policy item evaluations.

## Abbreviations

ARI: adjusted Rand index
BART: bidirectional and auto-regressive transformer
BERT: bidirectional encoder representations from transformers
BIC: Bayesian information criterion
BSS/WSS: between-cluster sum of squares to within-cluster sum of squares
CAPTA: Child Abuse Prevention and Treatment Act
CH: Calinski-Harabasz score
DeBERTa: decoding-enhanced BERT with disentangled attention
EFA: exploratory factor analysis
LLM: large language model
MRSF: multiple R-squared of scores with factors
NCANDS: National Child Abuse and Neglect Data System
NDACAN: National Data Archive on Child Abuse and Neglect
NLP: natural language processing
RoBERTa: robustly optimized BERT approach
SCAN: State Child Abuse & Neglect Policies Database
STROBE: Strengthening the Reporting of Observational Studies in Epidemiology

## Data Availability

The datasets used in this study are available from the National Data Archive on Child Abuse and Neglect (NDACAN) but are not publicly available due to data use restrictions. Both the National Child Abuse and Neglect Data System (NCANDS) and the State Child Abuse & Neglect Policies (SCAN) dataset require submission of a data use agreement and approval by NDACAN. Researchers may request access through NDACAN [66] and must comply with all data security and use requirements. Demographic and economic data from the American Community Survey are publicly available through the US Census Bureau.
