# Supplementary material for: Using Natural Language Processing to Examine State Child Maltreatment Policies and Associations With Outcomes: Multistate Cross-Sectional Study
Source: JMIR Public Health Surveill. 2026 Sep 16;12:e99643. doi: 10.2196/99643 (PMC13628039; doi:10.2196/99643)
Supplement: Supplementary file 1 [file publichealth_v12i1e99643_app1.docx]

Figure S1. Scree plot to determine the number of factors

Figure S2. Silhouette plot to determine the number of clusters

Figure S3. Factor scores heatmap by cluster

Figure S4. Percent below poverty density comparison

Figure S5. Pairwise outcome comparisons using all screened-in NCANDS maltreatment records in 2021

Figure S6. Sub-analysis clusters on the U.S. map

Table S1. List of 2021 SCAN policy items

| Policy Item Name | Policy Item Description |
| --- | --- |
| Def_PhysicalAbuse | Definition includes physical abuse |
| Def_Punishment | Definition includes corporal punishment or severe discipline |
| Def_SexAbuse | Definition includes sexual abuse or sexual exposure |
| Def_Emotional | Definition includes emotional maltreatment, moral neglect or |
| Def_Neglect | Definition includes neglect |
| Def_InadequateClothing | Definition includes inadequate clothing |
| Def_InadequateShelter | Definition includes inadequate shelter |
| Def_Malnourishment | Definition includes malnourishment |
| Def_MedicalNeglect | Definition includes medical neglect |
| Def_FailuretoThrive | Definition includes failure to thrive |
| Def_EducationalNeglect | Definition includes educational neglect |
| Def_Abandonment | Definition includes abandonment |
| Def_InjuriousEnvironment | Definition includes injurious environment |
| Def_DrugLab | Definition includes exposure to drug lab |
| Def_InadequateSupervision | Definition includes inadequate supervision or failure of |
| Def_DrugAlcMisuse | Definition includes drug or alcohol misuse |
| Def_PrenatalExposure | Definition includes prenatal exposure to drugs or alcohol |
| Def_IllicitSubstance | Definition includes illegal substance |
| Def_HumanTrafficking | Definition includes human trafficking or sexual exploitation |
| Def_GenitalMutilation | Definition includes genital mutilation |
| Def_ShakenBaby | Definition includes abusive head trauma or shaken baby |
| Def_FailureToProtect | Definition includes failure to protect |
| Def_DV | Definition includes exposure to domestic violence |
| Def_FactitiousDisorder | Definition includes Factitious Disorder/Munchausen Syndrome |
| Def_InstitutionalAbuseNeglect | Definition includes Institutional Abuse/Neglect |
| Def_Other | Definition includes other type of child maltreatment |
| Def_OtherSpecify | Definition includes other type of child maltreatment-specify |
| Def_Subtype_Abuse | Subtypes of maltreatment considered abuse |
| Def_Subtype_Neglect | Subtypes of maltreatment considered neglect |
| Def_Subtype_Other | Subtypes of maltreatment considered other maltreatment |
| DefMal_InflictHarm | Definition includes inflicting harm |
| DefMal_RiskOfHarm | Definition includes or substantial risk of harm or imminent danger |
| DefMal_Harm_VaryByType | Definition re inflicting/risk of harm varies by maltreatment type |
| DefMal_Harm_VaryByTypeSpecify | Definition of harm varies by maltreatment type-specify |
| DefHarm_Physical | Type of harm-physical harm |
| DefHarm_Emotional | Type of harm-emotional harm |
| DefHarm_Environment | Type of harm-harmful environment or conditions |
| DefHarm_NotSpecified | Type of harm-type of harm or injury not specified |
| DefHarm_Other | Type of harm-other |
| DefHarm_OtherSpecify | Type of harm specified-other specify |
| DefHarm_VaryByType | Definition re type of harm varies by maltreatment type |
| DefHarm_VaryByTypeSpecify | Definition of type of harm varies by maltreatment type-specify |
| DefPerp | Definition includes type of perpetrator |
| DefPerp_Parent | Definition includes perpetrator-parent |
| DefPerp_Guardian | Definition includes perpetrator-guardian |
| DefPerp_Caregiver | Definition includes perpetrator-caregiver |
| DefPerp_FamilyMember | Definition includes perpetrator-family member |
| DefPerp_HouseholdMember | Definition includes perpetrator-household member |
| DefPerp_PersonResponsible | Definition includes perpetrator-person responsible for child |
| DefPerp_AnyAdult | Definition includes perpetrator-any adult |
| DefPerp_Other | Definition includes perpetrator-other |
| DefPerp_OtherSpecify | Definition includes perpetrator-other specify |
| DefPerp_VaryByType | Definition of perpetrator varies by maltreatment type |
| DefPerp_VaryByTypeSpecify | Definition of perpetrator varies by maltreatment type-specify |
| DefAge | Definition specifies age of child |
| DefAge_Specify | Age of child specified in definition |
| DefAge_VaryByType | Age of child in definition varies by maltreatment type |
| DefAge_VaryByTypeSpecify | Age of child specified in definition varies by maltreatment type- |
| Exempt_FinancialIssues | Exemption from definition-financial issues |
| Exempt_Discipline | Exemption from definition-reasonable discipline |
| Exempt_SafeHaven | Exemption from definition-safe haven |
| Exempt_SubstanceExposed | Exemption from definition-substance exposed infant from |
| Exempt_Religious | Exemption from definition-religious medical treatment |
| Exempt_Other | Exemption from definition-other |
| Exempt_OtherSpecify | Exemption from definition-other specify |
| SafeHaven_Location | Safe haven exemption requires child be left at specific place or |
| SafeHaven_Parent | Safe haven exemption requires child be left by parent or parent's |
| SafeHaven_Age | Safe haven exemption requires child be left by certain age |
| SafeHaven_AgeSpecify | Safe haven exemption requires child be left by certain age- |
| SafeHaven_NoIntentReturn | Safe haven exemption requires no intent to return for child |
| SafeHaven_ChildUnharmed | Safe haven exemption requires child be left unharmed |
| SafeHaven_Other | Safe haven exemption requirement-other |
| SafeHaven_OtherSpecify | Safe haven exemption requirement-other specify |
| FatalitiesDefine_Not | Child fatalities not defined |
| FatalitiesDefine_Injury | Child fatalities definition includes injury |
| FatalitiesDefine_Abuse | Child fatalities definition includes abuse or neglect as |
| FatalitiesDefine_Care | Child fatalities definition includes death of children in foster care |
| FatalitiesDefine_Other | Child fatalities definition includes other |
| FatalitiesDefine_OtherSpecify | Child fatalities definition includes other - specify |
| FatalitiesDefine_Unknown | Child fatalities definition is unknown |
| FatalitiesReview | Child fatality case review |
| FatalitiesReview_OtherSpecify | Child fatality case review - other specify |
| NearFatalities_Not | Child near-fatalities not defined |
| NearFatalities_General | Child near-fatalities definition includes general reference to |
| NearFatalities_Specific | Child near-fatalities definition includes specific injury or treatment |
| NearFatalities_SpecificSpecify | Child near-fatalities definition includes specific injury or |
| NearFatalities_Other | Child near-fatalities definition inludes other |
| NearFatalities_OtherSpecify | Child near-fatalities definition includes other - specify |
| NearFatalities_Unknown | Child near-fatalities definition is unknown |
| NearFatalitiesReview | Child near-fatalities case review |
| NearFatalitiesReview_OtherSpec | Child near-fatalities case review - other specify |
| CentralizedReporting | Centralized reporting |
| CentralizedReporting_Specify | Other type of reporting |
| DecentralizedReporting | How is reporting decentralized |
| DecentralizedReporting_Specify | How is reporting decentralized-other specify |
| ReportStandard_Known | Reporting standard-known abuse and neglect |
| ReportStandard_ReasonCause | Reporting standard-reasonable cause |
| UniversalMandatedReporting | Universal mandated reporting for all adults |
| Reporter_FosterParent | Mandated reporter-foster parents |
| Reporter_TeacherSchool | Mandated reporter-school teachers |
| Reporter_BusDriver | Mandated reporter-school bus drivers and transportation staff |
| Reporter_AfterSchool | Mandated reporter-before or after school program staff |
| Reporter_ChildcareStaff | Mandated reporter-child care staff |
| Reporter_CampStaff | Mandated reporter-camp counselors |
| Reporter_Coach | Mandated reporter-athletic coaches or sports staff |
| Reporter_MedicalDental | Mandated reporter-medical or dental professionals |
| Reporter_SUDProvider | Mandated reporter-substance use disorder treatment providers |
| Reporter_MHProf | Mandated reporter-mental health and other social service |
| Reporter_Police | Mandated reporter-police or other law enforcement |
| Reporter_EMTEmergency | Mandated reporter-emergency medical technician |
| Reporter_Judges | Mandated reporter-judges |
| Reporter_DAAttorneys | Mandated reporter-district attorneys |
| Reporter_GALCASA | Mandated reporter-guardian ad litems |
| Reporter_OtherCourt | Mandated reporter-other court personnel |
| Reporter_ShelterStaff | Mandated reporter-shelter staff |
| Reporter_Images | Mandated reporter-those who process or monitor images |
| Reporter_Clergy | Mandated reporter-religious clergy |
| Reporter_Volunteer | Mandated reporter-volunteers |
| Reporter_Coroner | Mandated reporter- coroner or medical examiner |
| Reporter_StateCountyAgency | Mandated reporter-state and county agencies |
| Reporter_Other | Mandated reporter-other court personnel |
| Reporter_OtherSpecify | Mandated reporter-other specify |
| ReporterTrainingReq | Training required for mandated reporters |
| ReporterTraining_FosterParent | Reporter training-foster parents |
| ReporterTraining_TeacherSchool | Reporter training-school teachers |
| ReporterTraining_BusDriver | Reporter training-school bus drivers and transportation staff |
| ReporterTraining_AfterSchool | Reporter training-before or after school program staff |
| ReporterTraining_ChildcareStaff | Reporter training-child care staff |
| ReporterTraining_CampStaff | Reporter training-camp counselors |
| ReporterTraining_Coach | Reporter training-athletic coaches or sports staff |
| ReporterTraining_MedicalDental | Reporter training-medical or dental professionals |
| ReporterTraining_SUDProvider | Reporter training-substance use disorder treatment providers |
| ReporterTraining_MHProf | Reporter training-mental health and other social service |
| ReporterTraining_Police | Reporter training-police or other law enforcement |
| ReporterTraining_EMTEmergency | Reporter training-emergency medical technician |
| ReporterTraining_Judges | Reporter training-judges |
| ReporterTraining_DAAttorneys | Reporter training-district attorneys |
| ReporterTraining_GALCASA | Reporter training-guardian ad litems |
| ReporterTraining_OtherCourt | Reporter training-other court personnel |
| ReporterTraining_ShelterStaff | Reporter training- shelter staff |
| ReporterTraining_Images | Reporter training-those who process or monitor images |
| ReporterTraining_Clergy | Reporter training-religious clergy |
| ReporterTraining_Volunteer | Reporter training-volunteers |
| ReporterTraining_Coroner | Reporter training- coroner or medical examiner |
| ReporterTraining_StateCntyAgency | Reporter training-state and county agencies |
| ReporterTraining_Other | Reporter training-other court personnel |
| ReporterTraining_OtherSpecify | Reporter training-other specify |
| ReporterPenalty | Penalties for failure to report abuse and neglect |
| ReporterPenalty_FosterParent | Failure to report penalties-foster parents |
| ReporterPenalty_TeacherSchool | Penalties-school teachers |
| ReporterPenalty_BusDriver | Failure to report penalties-school bus drivers and transportation |
| ReporterPenalty_AfterSchool | Failure to report penalties-before or after school program staff |
| ReporterPenalty_ChildcareStaff | Failure to report penalties-child care staff |
| ReporterPenalty_CampStaff | Penalties-camp counselors |
| ReporterPenalty_Coach | Failure to report penalties-athletic coaches or sports staff |
| ReporterPenalty_MedicalDental | Failure to report penalties-medical or dental professionals |
| ReporterPenalty_SUDProvider | Failure to report penalties-substance use disorder treatment |
| ReporterPenalty_MHProf | Failure to report penalties-mental health and other social service |
| ReporterPenalty_Police | Failure to report penalties-police or other law enforcement |
| ReporterPenalty_EMTEmergency | Penalties-emergency medical technician |
| ReporterPenalty_Judges | Failure to report penalties-judges |
| ReporterPenalty_DAAttorneys | Penalties-district attorneys |
| ReporterPenalty_GALCASA | Penalties-guardian ad litems |
| ReporterPenalty_OtherCourt | Failure to report penalties-other court personnel |
| ReporterPenalty_ShelterStaff | Failure to report penalties-shelter staff |
| ReporterPenalty_Images | Failure to report penalties-those who process or monitor images |
| ReporterPenalty_Clergy | Failure to report penalties-religious clergy |
| ReporterPenalty_Volunteer | Failure to report penalties-volunteers |
| ReporterPenalty_Coroner | Failure to report penalties- coroner or medical examiner |
| ReporterPenalty_StateCntyAgency | Failure to report penalties-state and county agencies |
| ReporterPenalty_Other | Failure to report penalties-other court personnel |
| ReporterPenalty_OtherSpecify | Failure to report penalties-other specify |
| Penalty_Criminal | Failure to report penalty type-criminal penalties |
| Penalty_Civil | Failure to report penalty type-civil penalties |
| Penalty_Professional | Failure to report penalty type-professional |
| Penalty_Other | Failure to report penalties-other court personnel |
| Penalty_OtherSpecify | Failure to report penalties-other specify |
| FalseReportPenalty | Penalties for false reporting of abuse and neglect |
| FalseReportPenalty_Criminal | False reporting penalty type-criminal |
| FalseReportPenalty_Civil | False reporting penalty type-civil |
| FalseReportPenalty_Professional | False reporting penalty type-professional |
| FalseReportPenalty_Other | False reporting penalty type-other |
| FalseReportPenalty_OtherSpecify | False reporting penalty type-other specify |
| FalseReportPenalty_Unknown | False reporting penalty type-unknown |
| ReportImmunity | Immunity from penalties for reporters |
| ReportInfo_ChildIdentity | Requested report information-identifying and other information of |
| ReportInfo_ChildLocationContact | Requested report information-location/contact information-child |
| ReportInfo_Maltreatment | Requested report information-information about type of |
| ReportInfo_MaltreatmentDate | Requested report information-date of suspected maltreatment |
| ReportInfo_Perpetrator | Requested report information-identifying information-alleged |
| ReportInfo_Reporter | Requested report information-identifying and other information of |
| ReportInfo_Parent | Requested reporter information-parents, guardian, and |
| ReportInfo_FamilyMember | Requested reporter information-family/household members |
| ReportInfo_PriorMaltreatment | Requested reporter information-prior maltreatment of the child or |
| ReportInfo_Other | Required report information-other |
| ReportInfo_OtherSpecify | Required report information-other specify |
| ReportAnonymous | Can reporter remain anonymous |
| TribalReport | Tribes involved in accepting reports of tribal cases |
| TribalReport_Specify | Specify tribes involved in accepting reports |
| SENNotificationReport_No | All SENs cases are not required to be reported |
| SENNotificationReport_YesAll | All SENs cases are required to be reported |
| SENNotificationReport_Other | SENs cases required to be reported - other |
| SENNotificationReport_OtherSpec | Child sen-notification case report - other specify |
| SENNotificationReport_Unknown | Unknown if all SENs cares are required to be reported |
| RiskOnlyReport | Risk-only reports |
| RiskOnlyReport_OtherSpecify | Risk-only reports - other specify |
| CentralScreen | Centralized screening unit |
| DecentralScreen | How is screening decentralized |
| ScreenInfo_ChildName | Information required to screen report-child name |
| ScreenInfo_ChildAddr | Information required to screen report-child address |
| ScreenInfo_MalType | Information required to screen report-type of suspected |
| ScreenInfo_MalDate | Information required to screen report-date of suspected |
| ScreenInfo_Perp | Information required to screen report-identity of alleged |
| ScreenInfo_Other | Information required to screen report-other |
| ScreenInfo_OtherSpecify | Information required to screen report-other specify |
| ScreenInfo_Unknown | Information required to screen report-unknown |
| ScreenProcess_SupReview | Decision process for screening-supervisory review |
| ScreenProcess_Team | Decision process for screening-team based decision |
| ScreenProcess_Individual | Decision process for screening-individual screener |
| ScreenProcess_Other | Decision process for screening-other |
| ScreenProcess_OtherSpecify | Decision process for screening-other specify |
| ScreenProcess_Unknown | Decision process for screening-unknown |
| ScreenProcess_SupReviewReq | Screening process supervisory review required for cases |
| ScreenProcess_TeamReq | Screening process team-based decision for required cases |
| ScreenProcess_IndDecisionReq | Screening process individual decision required for cases |
| ScreenProcess_OtherReq | Screening process other decision process required for cases |
| ScreenProcess_Statewide | Screening decision process consistent statewide or vary locally |
| ScreenRequired | Activities or information required as part of screening |
| ScreenReq_RiskAssess | Required screening activity-safety or risk assessment |
| ScreenReq_PriorCPS | Required screening activity-Review records for prior CPS |
| ScreenReq_Other | Required screening activity-other |
| ScreenReq_OtherSpecify | Required screening activity-other specify |
| ScreenReq_RiskAssessCases | Safety or risk assessment for screening required for all/some |
| ScreenReq_RiskAssessCase_Specify | Specify cases that require safety or risk assessment for |
| ScreenReq_PriorCPSCases | Review of prior CPS records for screening required for all/some |
| ScreenReq_PriorCPSCases_Specify | Specify cases that require review of prior CPS records for |
| ScreenReq_OtherCases | Other activity or information for screening required for all/some |
| ScreenReq_OtherCases_Specify | Specify cases that require other activity or information for |
| ScreenRiskAssess_SDM | Safety or risk assessment tool used for screening-Structured |
| ScreenRiskAssess_Other | Safety or risk assessment tool used for screening-other |
| ScreenRiskAssess_OtherSpecify | Safety or risk assessment tool used for screening-other specify |
| ScreenInfo_Statewide | Screening activities/information consistent statewide or vary |
| ScreenInfo_VaryLocalSpecify | Screening activities/information vary locally-specify |
| Screener_Caseworker | Staff who conduct screening of reports-caseworker or frontline |
| Screener_Supervisor | Staff who conduct screening of reports-supervisor or managers |
| Screener_SpecialUnit | Staff who conducts screening of reports-specialized unit or staff |
| Screener_Other | Staff who conducts screening of reports-other |
| Screener_OtherSpecify | Staff who conducts screening of reports-other specify |
| ScreenerQual_Associate | Qualifications of staff who screen reports-associate's degree |
| ScreenerQual_Bachelor | Qualifications of staff who screen reports-bachelor's degree |
| ScreenerQual_Master | Qualifications of staff who screen reports-master's degree |
| ScreenerQual_Training | Qualifications of staff who screen reports-training |
| ScreenerQual_TrainingSpecify | Qualifications of staff who screen reports-training specify |
| ScreenerQual_Experience | Qualifications of staff who screen reports-years of experience |
| ScreenerQual_ExperienceSpecify | Specification of qualifications of staff who screen reports-years of |
| ScreenerQual_Other | Qualifications of staff who screen reports-other |
| ScreenerQual_OtherSpecify | Specification of qualifications of staff who screen reports-other |
| ScreenerQual_Unknown | Qualifications of staff who screen reports-unknown |
| ScreenerQual_SW | Qualifications of staff who screen reports-SW degree |
| ScreenerQual_SW_OtherSpecify | Specification of qualifications of staff who screen reports-SW |
| TribalScreen | Tribes involvement in screening of tribal cases |
| TribalScreen_Specify | Specify tribes involved in screening of tribal cases |
| InvPenalty_Criminal | Child maltreatment investigations lead can lead to criminal |
| InvestigationReq | Activities required as part of investigation process |
| InvestReq_PriorCPS | Required investigation activity-review records for prior CPS |
| InvestReq_HomeVisit | Required investigation activity-home visit |
| InvestReq_Victim | Required investigation activity-interview or observation of victim |
| InvestReq_OtherChild | Required investigation activity-interview or observation of other |
| InvestReq_RiskAssess | Required investigation activity-risk or safety assessment |
| InvestReq_HomeStudy | Required investigation activity-home study |
| InvestReq_Parent | Required investigation activity-interview of parents or caregivers |
| InvestReq_PerpCrim | Required investigation activity-check criminal records of |
| InvestReq_PerpPriorCPS | Required investigation activity-check prior CPS records of |
| InvestReq_MedEval | Required investigation activity-medical evaluation |
| InvestReq_MHEval | Required investigation activity-mental health evaluation |
| InvestReq_PerpInterview | Required investigation activity-interview alleged perpetrator |
| InvestReq_Reporter | Required investigation activity-interview reporter or collateral |
| InvestReq_Other | Required investigation activity-interview or observation of other |
| InvestReq_OtherSpecify | Required investigation activity-other specify |
| InvestReq_PriorCPSCases | Review of prior CPS records for investigation required for |
| InvestReq_PriorCPSCases_Specify | Specify cases that require prior CPS record review for |
| InvestReq_HomeVisitCases | Investigation requirement for home visit |
| InvestReq_HomeVisitCases_Specify | Specify cases that require home visit for investigation |
| InvestReq_VictimCases | Investigation requirement for victim interview |
| InvestReq_VictimCases_Specify | Specify cases that require victim interview for investigation |
| InvestReq_OtherChildrenCase | Investigation requirement for other child interview |
| InvestReq_OtherChildrenCase_Spec | Specify cases that require other children interviewed for |
| InvestReq_RiskAssessCases | Investigation requirement for risk assessment |
| InvestReq_RiskAssessCase_Specify | Specify cases that require risk assessment for investigation |
| InvestReq_HomeStudyCases | Investigation requirement for home study |
| InvestReq_HomeStudyCases_Specify | Specify cases that require home study for investigation |
| InvestReq_ParentCases | Investigation requirement for parent interview |
| InvestReq_ParentCases_Specify | Specify cases that require parent interview for investigation |
| InvestReq_PerpCrimCases | Investigation requirement for prior criminal record check |
| InvestReq_PerpCrimCases_Specify | Specify cases that require prior criminal record check for |
| InvestReq_PerpPriorCPSCases | Investigation requirement for central registry check |
| InvestReq_PerpPriorCPSCases_Spec | Specify cases that require central registry check for investigation |
| InvestReq_MedEvalCases | Investigation requirement for medical evaluation |
| InvestReq_MedEvalCases_Specify | Specify cases that require medical evaluation for investigation |
| InvestReq_MHEvalCases | Investigation requirement for mental health evaluation |
| InvestReq_MHEvalCases_Specify | Specify cases that require mental health evaluation for |
| InvestReq_PerpInterviewCases | Required investigation activity-interview alleged perpetrator for |
| InvestReq_PerpInterviewCase_Spec | Specify cases that require perpetrator interview for investigation |
| InvestReq_ReporterCases | Required investigation activity-interview reporter/sources for |
| InvestReq_ReporterCases_Specify | Specify cases that require reporter interview for investigation |
| InvestReq_OtherCases | Investigation requirement for other activity |
| InvestReq_OtherCases_Specify | Specify cases that require other activity for investigation |
| InvestStaff_Caseworker | Staff who conduct investigations of reports-case worker |
| InvestStaff_Supervisor | Staff who conduct investigations of reports-supervisor |
| InvestStaff_SpecialUnit | Staff who conduct investigations of reports-specialized unit or |
| InvestStaff_LawEnforcement | Staff who conduct investigations of reports-law enforcement |
| InvestStaff_Other | Staff who conduct investigations of reports-other |
| InvestStaff_OtherSpecify | Staff who conduct investigations of reports-other specify |
| InvestQual_Associate | Qualifications of investigation staff-associate's degree |
| InvestQual_Bachelor | Qualifications of investigation staff-bachelor's degree |
| InvestQual_Master | Qualifications of investigation staff-master's degree |
| InvestQual_Training | Qualifications of investigation staff-training |
| InvestQual_TrainingSpecify | Specification of qualifications of investigation staff-training |
| InvestQual_Experience | Qualifications of investigation staff-years of experience |
| InvestQual_ExperienceSpecify | Specification of qualifications of investigation staff-years of |
| InvestQual_Other | Qualifications of investigation staff-other |
| InvestQual_OtherSpecify | Qualifications of investigation staff-other specify |
| InvestQual_Unknown | Qualifications of investigation staff-unknown |
| InvestQual_SW | Qualifications of investigation staff-SW degree |
| InvestQual_SW_OtherSpecify | Specification of qualifications of investigation staff-SW degree |
| EvidenceLevel | Level of evidence required for substantiation |
| InvestInconclusive | Inconclusive investigation determination |
| AlternativeResponse | State has alternative or differential response |
| AlternativeResp_VaryLocalSpecify | Specification of how alternative response varies locally |
| AltResp_EligibilityMaltreatment | Eligibility for alternative response based on type of maltreatment |
| AltResp_Inelig_Fatality | Child fatality cases are not eligible for alternative response |
| AltResp_Inelig_SubExposed | Substance-exposed newborn cases are not eligible for |
| AltResp_Inelig_PhysicalAbuse | Physical abuse cases are not eligible for alternative response |
| AltResp_Inelig_SexAbuse | Sexual abuse cases are not eligible for alternative response |
| AltResp_Inelig_Neglect | Neglect cases are not eligible for alternative response |
| AltResp_Inelig_Abandoned | Abandoned infant cases are not eligible for alternative response |
| AltResp_Inelig_Other | Cases with other child maltreatment are not eligible for |
| AltResp_Inelig_OtherSpecify | Specification of other child maltreatment not eligible for |
| AltResp_EligibilityRisk | Eligibility for alternative response informed by risk determination |
| AltResp_EligibilityRisk_Specify | AltResp_EligibilityRisk_Specify |
| AltResp_EligibilityRiskTool | Tool used to determine risk for alternative response |
| AltResp_EligibilityRiskToolSpec | Tool is used to determine risk for alt response-specify |
| AltResp_EligibilityRisk_No | Risk level eligible for alternative response-no risk |
| AltResp_EligibilityRisk_Low | Risk level eligible for alternative response-low risk |
| AltResp_EligibilityRisk_Moderate | Risk level eligible for alternative response-moderate risk |
| AltResp_EligibilityRisk_Other | Risk level eligible for alternative response-other risk |
| AltResp_EligibilityRiskOtherSpec | Risk level eligible for alternative response-other specify |
| AltResp_EligibilityRisk_Unknown | Risk level eligible for alternative response-unknown |
| AltResp_Eligibility_NoOtherCases | Cases eligible for alternative response-no other conditions |
| AltResp_Eligibility_NoSafetyCon | Cases eligible for alternative response-no immediate safety |
| AltResp_Eligibility_PriorReports | Cases eligible for alternative response-no or few prior reports |
| AltResp_Eligibility_Other | Cases eligible for alternative response-other conditions |
| AltResp_Eligibility_OtherSpecify | Cases eligible for alternative response-other conditions specify |
| AltResp_Process_AtScreening | Alternative response determination process-at time of screening |
| AltResp_Process_AfterScreenIn | Alternative response determination process-after report |
| AltResp_Process_Other | Alternative response determination process-other |
| AltResp_Process_OtherSpecify | Alternative response determination process-other specify |
| AltResp_ServiceReferral_No | Alternative response cases do not have referrals to community |
| AltResp_ServiceReferral_AllCases | All alternative response cases have referrals to community |
| AltResp_ServiceReferral_Interest | Alternative response cases have referrals to community services |
| AltResp_ServiceReferral_Risk | Alternative response cases have referrals to community services |
| AltResp_ServiceReferralOther | Alternative response cases have referrals to community |
| AltResp_ServiceReferralOtherSpec | Alternative response cases have referrals to community |
| InHome_Unsubstantiated | In-home services provided for unsubstantiated cases |
| InHome_PostReunification | In-home services provided post reunification |
| TribalFoster | Tribes involved in providing foster care for tribal cases |
| TribalFoster_Specify | Specify tribes involved in providing foster care of tribal cases |
| ExtendedFosterCare | Extended foster care available for those older than 18 |
| ExtendedFosterCare_Age | Extended foster care available until age 21 |
| ExtendedFosterCare_Age_OtherSpec | Extended foster care available until what age-other specify |
| KinGuardianship | Kinship guardianship is a permanency option |
| SubGuardianship | State has subsidized guardianship |
| SubKinGuardianship | State has subsidized kinship guardianship |
| SubAdoption | State has subsidized adoption |
| CaseManagement_State | Staff who conduct foster care case management-state or county |
| CaseManagement_Provider | Staff who conduct foster care case management-contracted |
| CaseManagement_Tribal | Staff who conduct foster care case management-tribal agency |
| CaseManagement_Unknown | Staff who conduct foster care case management-unknown |
| CaseManagerQual_Associate | Qualifications of foster care case managers-associate's degree |
| CaseManagerQual_Bachelor | Qualifications of foster care case managers-bachelor's degree |
| CaseManagerQual_Master | Qualifications of foster care case managers-master's degree |
| CaseManagerQual_Training | Qualifications of foster care case managers-training |
| CaseManagerQual_TrainingSpecify | Qualifications of foster care case managers-training specify |
| CaseManagerQual_Experience | Qualifications of foster care case managers-years of experience |
| CaseManagerQual_ExperienceSpec | Qualifications of foster care case managers-years of experience |
| CaseManagerQual_Other | Qualifications of foster care case managers-other |
| CaseManagerQual_OtherSpecify | Qualifications of foster care case managers-other specify |
| CaseManagerQual_Unknown | Qualifications of foster care case managers-unknown |
| CaseManagerQual_SW | Qualifications of foster care case manager staff-SW degree |
| CaseManagerQual_SW_OtherSpec | Specification of qualifications of foster care case manager staff- |
| EquityStaffRole_No | State does not have equity staff roles |
| EquityStaffRole_CulturalBroker | State has cultural brokers |
| EquityStaffRole_Manager | State has DEI manager administrator or office |
| EquityStaffRole_Other | State has equity staff - other |
| EquityStaffRole_Unknown | State has equity staff - unknown |
| CulturalBroker_Screening | Cultural brokers are involved in screening |
| CulturalBroker_Investigation | Cultural brokers are involved in investigation |
| CulturalBroker_CaseMgmt | Cultural brokers are involved in case management or CW |
| CulturalBroker_Other | Cultural brokers are involved in process-other |
| CulturalBroker_OtherSpecify | Cultural brokers are involved in process-other specify |
| CulturalBroker_Unknown | Cultural brokers are involved in process-unknown |
| EquityStaff_Specify | Equity staff role or job funcation-specify |
| EquityStaff_Screening | Equity staff are involved in screening |
| EquityStaff_Investigation | Equity staff are involved in investigation |
| EquityStaff_CaseMgmt | Equity staff are involved in case management or CW response |
| EquityStaff_Other | Equity staff are involved in process-other |
| EquityStaff_OtherSpecify | Equity staff are involved in process-other specify |
| EquityStaff_Unknown | Equity staff are involved in process-unknown |
| CWSystemAdmin | State child welfare system is state/county-administered |
| ConsentDecree | State operates under a legal consent decree |
| ConsentDecree_Specify | State operates under legal consent decree-specify |

Table S2. Detailed sub-analysis of fatality-related policy item lists

Child fatalities not defined

Child fatalities definition includes injury

Child fatalities definition includes abuse or neglect as contributing factor

Child fatalities definition includes death of children in foster care

Child fatalities definition includes other

Child fatalities definition includes other - specify

Child fatalities definition is unknown

Child fatality case review

Child fatality case review - other specify

Child near-fatalities not defined

Child near-fatalities definition includes general reference to condition/injury

Child near-fatalities definition includes specific injury or treatment

Child near-fatalities definition includes specific injury or treatment-specify

Child near-fatalities definition includes other

Child near-fatalities definition includes other - specify

Child near-fatalities definition is unknown

Child near-fatalities case review

Child near-fatalities case review - other specify

Table S3. Qualitative review results

| Evaluation Type | Issue Observed | Reasons Based on Review | Example Errors & Observations |
| --- | --- | --- | --- |
| Intrinsic Eval – Category Consistency | RoBERTa had the lowest category consistency score | Struggles with negation, e.g., "does not", "failure to report" | Example: "Mandated reporters who are before or after school staff are subject to penalties for failure to report child abuse and neglect." RoBERTa misclassified this due to difficulty handling "failure to report." |
|  | Llama had a low category consistency score | Invents artificial logic | Example: "Definition includes educational neglect." Llama 3.1 created incorrect logic, reasoning that neglect isn’t in abuse definitions. |
| Intrinsic Eval – Semantic Alignment | There is substantial variability in the semantic alignment score for LLM models | Create artificial logic instead of following expected patterns | Example: "Investigation requirement for other child interview". Llama 3.1 estimated likelihoods with fabricated justifications rather than following the response structure. |
|  |  | Sometimes demonstrates better understanding of long descriptive text | Example: "How is reporting decentralized?" Copilot provided better-aligned likelihoods than DeBERTa in some of the longer response options. |
|  |  | Sometimes hard to determine which model is better | Semantic alignment was compared to similarity scores and average scores across methods, but these are imperfect proxies. |
| Comparison of Similarity & Average Score Approaches | DeBERTa is neither the best nor the worst in terms of intrinsic semantic alignment scores | DeBERTa makes more sense than the similarity score in some cases. Similarity score is not the best proxy standard for long texts | Example: "Specification of how screening varies locally". DeBERTa made more reasonable estimates for “Not Applicable”, while similarity scores tend to overestimate the score for these texts. |
|  |  | DeBERTa makes less sense than the average score in some cases, especially when processing long texts | Example: "Specification of other process that involves cultural brokers." Some details were better captured by the average score for this item. |
| Extrinsic Eval | DeBERTa shows the best results overall | DeBERTa likely better preserves key relationships or structures in the data, maintaining more meaningful variance. | Best scores on BIC, MRSF, CH, and BSS/WSS metrics, suggesting it preserved structural relationships better than other models. |

Table S4. Top 10 policy items for each factor from factor analysis

| Factor 1: Definition | Factor 2: Reporting | Factor 3: Alternative Responses |
| --- | --- | --- |
| Definition includes abandonment | Universal mandated reporting for all adults | Cases eligible for alternative response: no or few prior reports |
| Definition includes inadequate supervision or failure of responsibilities | Penalties for failure to report abuse and neglect | Eligibility for alternative response based on type of maltreatment |
| Definition includes inflicting harm | Failure to report penalties: state and county agencies | State has alternative or differential response |
| Type of harm: emotional harm | Mandated reporter: judges | Alternative response cases do not have referrals to community |
| Definition specifies child age | Failure to report penalties: judges | Child fatality cases are not eligible for alternative response |
| Exemption from definition: safe haven | Failure to report penalties: coroner or medical examiner | Sexual abuse cases are not eligible for alternative response |
| Safe haven exemption requires child be left at specific place or provider | **[N]** Reporter training: medical or dental professionals | Risk level eligible for alternative response: no risk |
| Safe haven exemption requires child be left by certain age | Failure to report penalties: those who process or monitor images | Eligibility for alternative response informed by risk determination |
| Definition includes sexual abuse or sexual exposure | Mandated reporter: those who process or monitor images | **[N]** Centralized reporting |
| Child near-fatalities definition is unknown | **[N]** Reporter training: coroner or medical examiner | **[N]** Centralized screening unit |

Note: items with **[N]** indicate that the loading was negative, meaning these items have an inverse relationship with the corresponding factor.

Table S5. Outcome comparison using all screened-in NCANDS maltreatment records in 2021

|  | Cluster 1  (n = 12) | Cluster 2  (n = 6) | Cluster 3  (n = 23) | Cluster 4  (n = 11) | p-value |
| --- | --- | --- | --- | --- | --- |
| Incidence of Maltreatment | 54.39 (17.11) | 66.76 (11.58) | 48.36 (23.72) | 61.16 (40.26) | 0.354 |
| Maltreatment Recurrence | 6.87 (3.22) | 10.34 (3.29) | 6.30 (3.73) | 7.08 (5.36) | 0.191 |
| Maltreatment Fatalities | 21.63 (14.83) | 25.74 (14.70) | 25.80 (15.64) | 23.20 (18.80) | 0.889 |

^a^ Values are presented as mean (SD). P-values are based on one-way ANOVA tests.

Table S6. Fatality-related policy item comparison among clusters based on 18 fatality-related policies

|  | Cluster 1  (n = 15) | Cluster 2  (n = 3) | Cluster 3  (n = 20) | Cluster 4  (n = 14) | p-value |
| --- | --- | --- | --- | --- | --- |
| Child fatalities not defined | 0.42 (0.00) | 0.42 (0.00) | 0.45 (0.08) | 0.42 (0.00) | 0.074 |
| Child fatalities definition includes injury | 0.64 (0.00) | 0.34 (0.00) | 0.55 (0.14) | 0.62 (0.08) | <0.001 |
| Child fatalities definition includes abuse or neglect as contributing factor | 0.72 (0.00) | 0.72 (0.00) | 0.08 (0.00) | 0.72 (0.00) | <0.001 |
| Child fatalities definition includes death of children in foster care | 0.22 (0.00) | 0.22 (0.00) | 0.29 (0.15) | 0.58 (0.00) | <0.001 |
| Child fatalities definition includes other | 0.43 (0.05) | 0.42 (0.00) | 0.48 (0.09) | 0.45 (0.07) | 0.257 |
| Child fatalities definition includes other - specify | 0.47 (0.01) | 0.47 (0.00) | 0.50 (0.07) | 0.49 (0.06) | 0.300 |
| Child fatalities definition is unknown | 0.36 (0.00) | 0.36 (0.00) | 0.36 (0.00) | 0.36 (0.00) | 0.562 |
| Child fatality case review | 0.69 (0.01) | 0.69 (0.00) | 0.69 (0.02) | 0.69 (0.00) | 0.614 |
| Child fatality case review - other specify | 0.48 (0.10) | 0.45 (0.00) | 0.47 (0.09) | 0.45 (0.00) | 0.797 |
| Child near-fatalities not defined | 0.44 (0.00) | 0.47 (0.06) | 0.47 (0.05) | 0.44 (0.03) | 0.057 |
| Child near-fatalities definition includes general reference to condition/injury | 0.57 (0.03) | 0.53 (0.07) | 0.54 (0.05) | 0.56 (0.03) | 0.180 |
| Child near-fatalities definition includes specific injury or treatment | 0.45 (0.07) | 0.42 (0.00) | 0.43 (0.05) | 0.44 (0.06) | 0.762 |
| Child near-fatalities definition includes specific injury or treatment-specify | 0.47 (0.01) | 0.47 (0.00) | 0.48 (0.02) | 0.48 (0.03) | 0.660 |
| Child near-fatalities definition includes other | 0.46 (0.00) | 0.46 (0.00) | 0.47 (0.02) | 0.46 (0.00) | 0.672 |
| Child near-fatalities definition includes other - specify | 0.47 (0.00) | 0.47 (0.00) | 0.47 (0.02) | 0.47 (0.00) | 0.672 |
| Child near-fatalities definition is unknown | 0.45 (0.00) | 0.45 (0.00) | 0.45 (0.00) | 0.45 (0.00) | 0.562 |
| Child near-fatalities case review | 0.59 (0.05) | 0.58 (0.07) | 0.60 (0.05) | 0.61 (0.03) | 0.743 |
| Child near-fatalities case review - other specify | 0.45 (0.00) | 0.45 (0.00) | 0.45 (0.02) | 0.45 (0.00) | 0.672 |
